# Effect of hypnotic suggestion on cognition and craving in smokers^☆^

**DOI:** 10.1016/j.abrep.2019.100220

**Published:** 2019-11-26

**Authors:** J.W. Bollinger, C.W. Beadling, A.J. Waters

**Affiliations:** a Department of Medical and Clinical Psychology, Uniformed Services University of the Health Sciences, Bethesda, MD, United States; b Department of Military and Emergency Medicine, Uniformed Services University of the Health Sciences, Bethesda, MD, United States

**Keywords:** Hypnosis, Hypnotic suggestion, Cigarette craving, Classic Stroop, Smoking Stroop

## Abstract

Cigarette smoking remains an important public health concern, and novel smoking cessation interventions are needed. Craving for cigarettes is a well-established target for intervention. Recent research has identified cognitive processes associated with craving, such as attentional bias to smoking cues, as targets for intervention. One intervention that may be effective in reducing attentional bias is hypnotic suggestion. Using a counterbalanced within-subjects design in a sample of active smokers (*N* = 33), this laboratory study examined if hypnotic suggestion can modify attention, assessed by the classic and smoking Stroop tasks, and craving. Hypnotic susceptibility was assessed using the Stanford Scale of Hypnotic Susceptibility. There was no evidence that hypnotic susceptibility moderated the effect of hypnotic suggestion on the classic or smoking Stroop effects. However, hypnotic susceptibility did moderate the effect of hypnotic suggestion on craving, such that hypnotic suggestion reduced craving in individuals with high levels of susceptibility. Further research into the mechanisms by which hypnotic suggestion reduces craving is warranted.

## Highlights

• Study examined effect of hypnotic suggestion on attention and craving for cigarettes.
• Effect of hypnotic suggestion on craving was moderated by level of hypnotic susceptibility.
• Hypnotic suggestion reduced craving in individuals with higher hypnotic susceptibility.

## 1 Introduction

Cigarette smoking remains the leading cause of preventable death in the United States (Office of the Surgeon General, 2014). Despite the availability of efficacious smoking cessation treatments, most quit attempts end in relapse (Centers for Disease Control and Prevention, 2011). Therefore, novel smoking cessation interventions are needed.

Recent research has revealed a number of cognitive targets for intervention (Robinson, Waters, Kang, & Sofuoglu, 2017). For example, research has examined cognitive biases associated with smoking, such as attentional bias to smoking cues (Rooke, Hine, & Thorsteinsson, 2008). Some research has reported that attentional bias is associated with craving (Field, Munafo, & Franken, 2009) and poor smoking cessation outcomes (Christiansen et al., 2015, Janes et al., 2010).

One task used to assess attentional bias to smoking cues is the smoking Stroop task, a variant of the classic Stroop task. In the classic Stroop task, individual words are presented one after the other in different colored text in the center of a computer screen. Participants are required to identify the color of the word as quickly as possible. Typically, participants are slower to identify the colors of words on “incongruent” stimuli (e.g., the word GREEN written in red ink) in comparison to “congruent” stimuli (e.g., the word RED written in red ink) (MacLeod, 1991). Following Raz and Campbell (2011), the classic Stroop effect can be defined as the difference in reaction times between incongruent and congruent stimuli. In the smoking Stroop task, participants are required to idenitfy the colors of smoking words (e.g., CIGARETTE) and neutral words (e.g., FURNITURE). The smoking Stroop effect, a measure of attentional bias to smoking cues, is defined as the difference in reaction times to identify colors of smoking related words and neutral words (Cox, Fadardi, & Pothos, 2006).

Interventions, such as cognitive bias modification (Attwood, O'Sullivan, Leonards, Mackintosh, & Munafò, 2008) have been developed to target cognitive processes. However, the beneficial effects of cognitive bias interventions on abstinence have been modest or inconsistent (Cristea, Kok, & Cuijpers, 2016; but see Boffo et al., 2019, Rinck et al., 2018). Another intervention type that can target cognitive processes is hypnotic suggestion. Hypnosis can be defined as “a state of consciousness involving focused attention and reduced peripheral awareness characterized by an enhanced capacity for response to suggestion” (Division 30 Executive Committee, 2014). Hypnosis involves three phases, induction, deepening, & suggestion. During induction, the participant is asked to pay attention to a cue in their internal or external environment. During deepening, the participant is instructed to become immersed in the hypnotic state, usually by way of a countdown. During suggestion the participant is given instructions to experience specific changes in physical or psychological sensations. Most pertinent to the current study, several studies have reported that hypnotic suggestion improves attentional performance on the classic Stroop task, as well as other cognitive tasks (Augustinova and Ferrand, 2012, Iani et al., 2009, Iani et al., 2006, Parris and Dienes, 2013, Parris et al., 2014, Raz and Campbell, 2011, Raz et al., 2003, Raz et al., 2006, Raz et al., 2007, Raz et al., 2002, Terhune et al., 2017, Virta et al., 2015).

From a cognitive perspective, hypnotic suggestion has been described as a “unique form of top-down regulation” that can diminish the power of task irrelevant distractors to disrupt performance (Terhune et al., 2017). It does not elicit better cognitive control in itself; rather it may obviate the need for cognitive control (Terhune et al., 2017). Although the effect of hypnotic suggestion on cognitive processes associated with smoking has not yet been examined, theory suggests hypnotic suggestion may improve (through top-down regulation) a smoker’s ability to ignore task-irrelevant smoking-related stimuli. Importantly, some studies reported that psychotherapy involving hypnotic suggestions promoted smoking cessation (Carmody et al., 2008, Spiegel et al., 1993). However, in aggregate the results of controlled trials involving hypnosis have been inconclusive, perhaps due to variability in methodology of suggestion-based interventions used (Barnes et al., 2010).

Craving for cigarettes is another target for intervention (Herd, Borland, & Hyland, 2009). Few studies have examined the acute effect of hypnotic suggestion on craving, and none have done so using suggestions targeting cognition. One study reported that aversive conditioning reduced craving in smokers (Li et al., 2017). Li et al. used a “disgust” suggestion - smokers were told that cigarettes would smell and taste like excrement. However, one should note a methodological limitation of the Li et al. study: all participants completed a baseline (control) condition before the suggestion condition, meaning that completion of conditions (baseline vs. suggestion) was not counterbalanced over participants. The study reported in this paper used a counterbalanced within-subjects design, whereby each participant completed all assessments twice, after no suggestions and under normal conditions (hereafter referred to as the “control condition”), and after hypnotic suggestions (“hypnotic suggestion condition”).

Based on theory and data (Raz et al., 2002, Raz et al., 2005), it was predicted that effects of hypnotic suggestion would be moderated by degree of hypnotic susceptibility, which refers to a participant’s relative ability to experience and change behaviors/cognitions during hypnosis (Division 30 Executive Committee, 2014). In the current study, hypnotic susceptibility was assessed with the Stanford Hypnotic Susceptibility Scale, Form C (SHSS:C).

In sum, the aims of the study were to examine if hypnotic suggestion reduces the classic Stroop effect and the smoking Stroop effect particularly in participants with high levels of hypnotic susceptibility. The study also examined the effect of hypnotic suggestion on craving.

## 2 Methods

### 2.1 Participants

Participants were 33 adult smokers recruited from the Washington, DC metro area. The inclusion criteria were: (1) Aged 18–65; (2) Reported smoking at least 5 cigarettes/day; (3) Had a home address and a functioning telephone number; (4) Specified English as the first language. The exclusion criteria were: (1) Self-reported color vision deficiency; (2) Indicated a history of being diagnosed with schizophrenia, bipolar disorder, psychosis, a personality disorder, or PTSD; (3) Reported any “recent major life changes.“ Exclusion criteria #2 and #3 were based on Jensen’s (2011) manual which identified participants who may be at risk of experiencing anxiety during hypnosis. Participants received up to $100 for completing the single laboratory visit, which lasted approximately 120 min.

### 2.2 Procedure

The following procedures were approved by Uniformed Services University of the Health Sciences’ Institutional Review Board. During phone screening, participants were given a description of the study and provided verbal agreement to be screened. Eligible participants were scheduled for a single in-person laboratory session. In total, 64 persons were screened, 51 were eligible, and 33 attended and completed the study session. Upon arrival, the research assistant provided a detailed description of the study and obtained written informed consent. Mean visit time was 1:38 PM (range = 10:00 AM to 5:30 PM). Participants provided an expired breath sample for assessment of carbon monoxide. Participants then completed computer-administered questionnaires assessing demographics, recent smoking, degree of nicotine dependence (Fagerström Test for Nicotine Dependence; FTND, Heatherton, Kozlowski, Frecker, & Fagerström, 1991), smoking urges (Questionnaire of Smoking Urges-Brief; QSU-B, Cox, Tiffany, & Christen, 2001), perceptions of hypnosis (described later), and side effects (also described later).

**Fig. 1:**
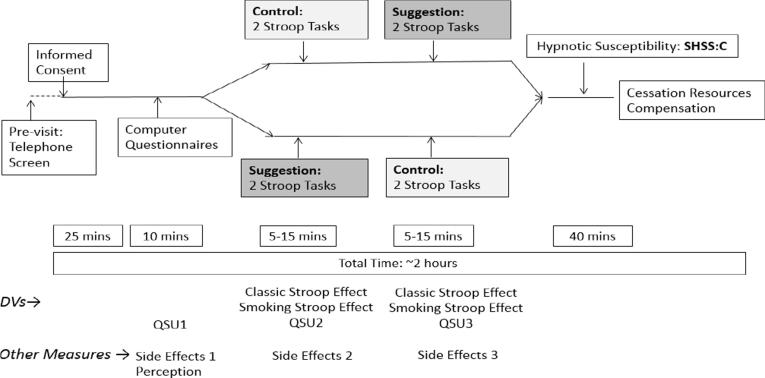
Study diagram.

Each participant completed assessments at both levels of Suggestion with order counterbalanced (see Fig. 1). This within-subjects design (for Suggestion) is consistent with the approach of past studies investigating the effect of hypnotic suggestion using the classic Stroop task (Raz et al., 2005, Raz et al., 2002). Sixteen participants completed the Suggestion-Control order and 17 the Control-Suggestion order. Before the Control condition, participants were not given hypnotic suggestions and were instructed to complete the task under normal conditions. Before the hypnotic suggestion condition, participants were read a scripted introduction to hypnosis adapted from the Harvard Group Scale of Hypnotic Susceptibility; HGSHS (Shor & Orne, 1962). Provision of an introduction to hypnosis is recommended as a means of decreasing any possible hypnotic sequelae (Crawford et al., 1982, Page and Green, 2002). In addition, a post-hypnosis “interview” was conducted to ensure hypnosis was no longer being experienced (Page & Green, 2002).

All participants completed the classic Stroop task before the smoking Stroop task. After each classic/smoking Stroop administration, participants completed the QSU and questions on side effects.

The Stanford Hypnotic Susceptibility Scale, Form C (SHSS:C) was administered after completion of both the Suggestion and Control conditions (Fig. 1).

At the end of the experiment, each participant was offered local, state, and national resources on smoking cessation programs (Fig. 1), thanked for their participation, and informed as to how they would receive compensation.

#### 2.2.1 Hypnotic suggestion condition

The hypnotic suggestion condition used the same suggestions described in Raz et al., 2002, Raz et al., 2005. The suggestions were preceded by a hypnotic induction and deepening from the SHSS:C manual (see Supplementary Materials, Fig. S1). The script for the suggestions was as follows: “Very soon you will be playing the computer game. When I clap my hands, meaningless symbols will appear in the middle of the screen. They will feel like characters of a foreign language that you do not know, and you will not attempt to attribute any meaning to them. This gibberish will be printed in one of 4 ink colors: red, blue, green or yellow. Although you will only be able to attend to the symbols' ink color, you will look straight at the scrambled signs and crisply see all of them. Your job is to quickly and accurately depress the key that corresponds to the ink color shown. You will find that you can play this game easily and effortlessly. ” (Raz et al., 2002). An additional phrase was added to this script based on Zahedi, Stuermer, Hatami, Rostami, and Sommer (2017): “When I clap my hands twice, you will regain your normal reading abilities.” Upon completion of the classic and smoking Stroop and QSU, the participant heard the following script from the experimenter, to ensure that participants were fully de-hypnotized “Close your eyes. I will now count down from 20 to 1: when 5 has been reached, you will open your eyes but you will be very slightly hypnotized; when I say 1, you will return to the here and now, and be completely alert and awake, and no longer hypnotized at all” (Zahedi et al., 2017).

The suggestions included a signal (double clap) to cancel the suggestions after the completion of the task (Fig. S1). The experimenter tested the suggestions had been cancelled by asking the participant to identify three colored words. All participants successfully completed this test.

#### 2.2.2 Control condition

In the control condition, participants did not undergo a hypnotic induction and deepening, and were not given suggestions. Participants performed the classic and smoking Stroop tasks under “normal” conditions as described later.

### 2.3 Measures

#### 2.3.1 Biochemical measure

Carbon monoxide (CO) was assessed using the Bedfont Micro^+^ smokelyzer system (Bedfont Scientific, Maidstone, Kent).

#### 2.3.2 Self-report assessments

The total score from the 10-item QSU-B (Cox et al., 2001) assessed craving “right now”. The QSU-B was administered via computer after completion of the classic and smoking Stroop at both levels of Suggestion (Fig. 1). The QSU-B has good reliability (e.g., Toll, Katulak, & McKee, 2006), and its validity has been established through cigarette cue exposure experimental paradigms (Cox et al., 2001). The 6-item Fagerström Test for Nicotine Dependence (FTND; Heatherton et al., 1991) assessed nicotine dependence (Appendix B). Higher scores indicate higher levels of dependence. The attitudes towards using hypnosis questionnaire was adapted and modified from an assessment used by Miller, Schnur, Montgomery, and Jandorf (2011). The assessment used comprised four items (each 0–10 scale) with higher scores indicating greater interest in using hypnosis for smoking cessation during a future quit attempt. A 3-item author constructed questionnaire assessed side effects: drowsiness, confusion, and headache (0–4 scales: 0 = “not at all”, 1 = “a little”, 2 = “somewhat”, 3 = “a lot”, 4 = “very much”). Side effects were assessed at baseline, after the control condition, and after the hypnotic suggestion condition.

#### 2.3.3 Classic & smoking Stroop

The classic and smoking Stroop tasks were implemented using e-Prime software (Psychology Software Tools, Pittsburgh, PA). The instructions informed participants that words would appear in different colored fonts on the computer screen, one after the other, and that their task was to indicate as rapidly and as accurately as possible which color the word was written in by pressing one of four response buttons on the computer keyboard. Specific computer keys corresponded to colors (e.g., V key for yellow, B key for blue, N key for green, M key for red; these keys had stickers with corresponding colors on the top of each key). Participants were instructed they should ignore the meaning of the (target) word itself and to respond to the color. A practice version involving 33 trials of meaningless letter strings (e.g., HHHH) was administered before the Stroop tasks.

Participants sat at a viewing distance of approximately 65 cm from the computer monitor. At the outset of each trial, a black fixation cross was presented for 1 s in the center of the computer screen. Each trial’s stimulus (i.e., a written word) was a single word displayed in one of four colors (red, blue, green, yellow) appearing at the center of the computer screen. All characters were displayed in upper case font against a white background; the stimuli subtended a visual angle of ~0.5° vertically, and 1.3° to 1.9° horizontally (depending on word length). The task for the subject was to identify the color in which the word is written by pressing a button corresponding to that color. Five-hundred milliseconds after a button-press response (or 500 ms after a 3-s timeout), a new word was presented. During the inter-trial interval, the screen was blank.

For the classic Stroop task, three classes of words were used: (1) congruent words (RED, BLUE, GREEN, and YELLOW) written in corresponding red, blue, green, and yellow font color; (2) incongruent words (RED, BLUE, GREEN, and YELLOW) in which the colors and words are mismatched, e.g., GREEN written in red font color; and (3) neutral words (LOT, SHIP, KNIFE, and FLOWER) used by Raz et al. (2002). The neutral words were length- and frequency-matched to the color words. There were 44 trials of congruent, incongruent, and neutral words, respectively, for a total of 132 trials during the classic Stroop task which took approximately six minutes to complete.

For the smoking Stroop task, two classes of words were used: (1) Smoking words, and (2) Neutral words. We used 11 smoking words (PACK, URGE, PUFF, DRAG, SMOKE, TOBACCO, ASHTRAY, CRAVING, NICOTINE, CIGARETTE, INHALE) used by Waters et al. (2003), and 11 matched neutral words (SINK, IRON, SOFA, COUCH, CARPET, LOUNGE, CURTAIN, HALLWAY, BOOKCASE, FURNITURE, TIDY). The word sets were of comparable length (5.91 vs 5.81 letters, respectively) and frequency (13.5 vs 15.3, respectively). There were 33 trials of Smoking and Neutral words, respectively, for a total of 66 trials for the smoking Stroop task which took approximately 70 s to complete.

#### 2.3.4 Hypnotic susceptibility

A structured induction and deepening was used for assessment of hypnotic susceptibility. After the induction and deepening, the participant was rated on 10 progressively difficult behavioral tasks. The Stanford Scale of Hypnotic Susceptibility, Form C (SHSS:C; Weitzenhoffer & Hilgard, 1962) was scored in this study using a 0–10 scale (item #7 [age regression] items and item #9 [anosmia to ammonia] were omitted to reduce potential for adverse events; Raz et al., 2002, Raz et al., 2005). Scores from 8 to 10 were defined as “high” levels of susceptibility. There is evidence that the test-retest reliability of the SHSS:C is strong (Piccione, Hilgard, & Zimbardo, 1989). The internal reliability as reported in the SHSS:C manual is *r* = 0.85 as determined by the Kuder-Richardson formula (a special case of Cronbach’s alpha applied to binary data). Reliability is not significantly affected by reducing or abbreviating the scale (Weitzenhoffer & Hilgard, 1962). The SHSS:C correlates significantly with several other scales of hypnotic susceptibility (Hilgard, 1978), and it is also used as a “gold-standard” comparison when other susceptibility scales are developed (Kekecs, Bowers, Johnson, Kendrick, & Elkins, 2016). Ratings from the SHSS:C also correlate with subjective/clinical ratings of susceptibility (O'Connell & Orne, 1966). Multiple studies have also shown that degree of hypnotic susceptibility is correlated with smoking abstinence after suggestion-based interventions (Perry and Mullen, 1975, Schubert, 1983, Barabasz et al., 1986, Spiegel et al., 1993). In other words, participants with higher scores on the SHSS:C tend to benefit more from suggestion-based interventions. Relevant to the current study, hypnotic suggestion reduces the classic Stroop effect in high susceptibility groups only.

### 2.4 Data analysis

The independent variables were Susceptibility (continuous variable), and Suggestion (a within-subject categorical variable with 2 levels, hypnotic suggestion vs. control). The primary dependent variables were the classic and smoking Stroop effects (see Section S.1 in Supplementary Materials for detail on scoring), and QSU ratings. For primary analyses, a general linear model (SAS PROC GLM) was used to determine the effect of Susceptibility and Suggestion condition on the classic Stroop and smoking Stroop effects (see Section S.2 in Supplementary Materials for power analysis). No demographic or smoking variables were significantly associated with Susceptibility (see Section S.3 in Supplementary Materials for consideration of covariates). Order (hypnotic suggestion condition first vs. control condition first) was included as a between-subjects independent variable. Order did not interact with Susceptibility in any analyses, and so Order × Susceptibility interactions were dropped from models (see section S.4 in Supplementary Materials for additional analyses involving Order).

## 3 Results

Participants were on average 50.70 years old (*SD* = 9.78), and 54.55% were female. The majority of the participants (78.79%) self-identified as Black. The total sample reported smoking an average of 13.18 cigarettes per day (*SD* = 7.96) (see Table 1 for more detail on participants).

**Table 1:** Participant characteristics.

***Variable ↓***	***Mean*/%**	***SD***
**Age** (years)	50.70	9.78
**Education** (number of years)	13.48	1.94
**Gender**		
Female	54.55%	
Male	45.45%	
**Marital Status**		
Single, never married	48.48%	
Married	21.21%	
Divorced	21.21%	
Widowed	3.03%	
Living with significant other	6.06%	
**Race**		
African American/Black	78.79%	
Mixed race	9.09%	
Anglo American/Euro American/White	6.06%	
Other	6.06%	
**Employment Status**		
Regular full time (30 + hours per week)	21.21%	
Regular part time (<30 h per week)	18.18%	
Unable to work or disabled	18.18%	
Unemployed, currently looking for work	12.12%	
Unemployed, NOT currently looking for work	12.12%	
Retired	9.09%	
Other	6.06%	
Student	3.03%	
**Income**		
Less than $10,000 per year	21.21%	
Between $10,000 and $19,999 per year	15.15%	
Between $20,000 and $29,999 per year	15.15%	
Between $30,000 and $39,999 per year	6.06%	
Between $40,000 and $49,999 per yea	15.15%	
Between $50,000 and $59,999 per year	12.12%	
Between $60,000 and $69,999 per year	6.06%	
Between $70,000 and $79,999 per year	6.06%	
Between $80,000 and $89,999 per year	3.03%	
**Main source of income**		
A Job	42.42%	
VA/disability/Social Security income	27.27%	
Other	15.15%	
A spouse/significant other/parent	9.09%	
Unemployment benefits	6.06%	
Cigarettes Smoked Per Day	13.18	7.96
FTND (0–10)	4.55	2.31
Expired breath CO level (ppm)	17.30	11.22
Perceptions (0–10)	30.59	6.43

Table Note: Mean (*SD*) (continuous measures) and % (categorical measures).

### 3.1 Descriptive statistics

Descriptive statistics for the SHSS:C were: *Mean* = 5.09, *Median* = 5.00, *SD* = 2.30, *Skew* = −0.30 (*SE* = 0.41), and *Kurtosis* = −0.59 (*SE* = 0.80). Four participants reported SHSS:C scores ≥ 8. Given the smaller than expected number of “high susceptible” individuals, all analyses that examined the effect of hypnotic suggestion on outcomes were also conducted using a cut-off of SHSS:C scores ≥ 7 (11 individuals). For the Perceptions of Hypnosis Questionnaire, the descriptive statistics (aggregated over 4 items) were: *Mean* = 7.64, *Median* = 7.50, *SD* = 1.58, *Skew* = −0.68 (*SE* = 0.41), and *Kurtosis* = 0.25 (*SE* = 0.80) (see section S.1 in Supplementary Materials for summary statistics on the Stroop tasks).

### 3.2 Effects of hypnotic suggestion

**Fig. 2:**
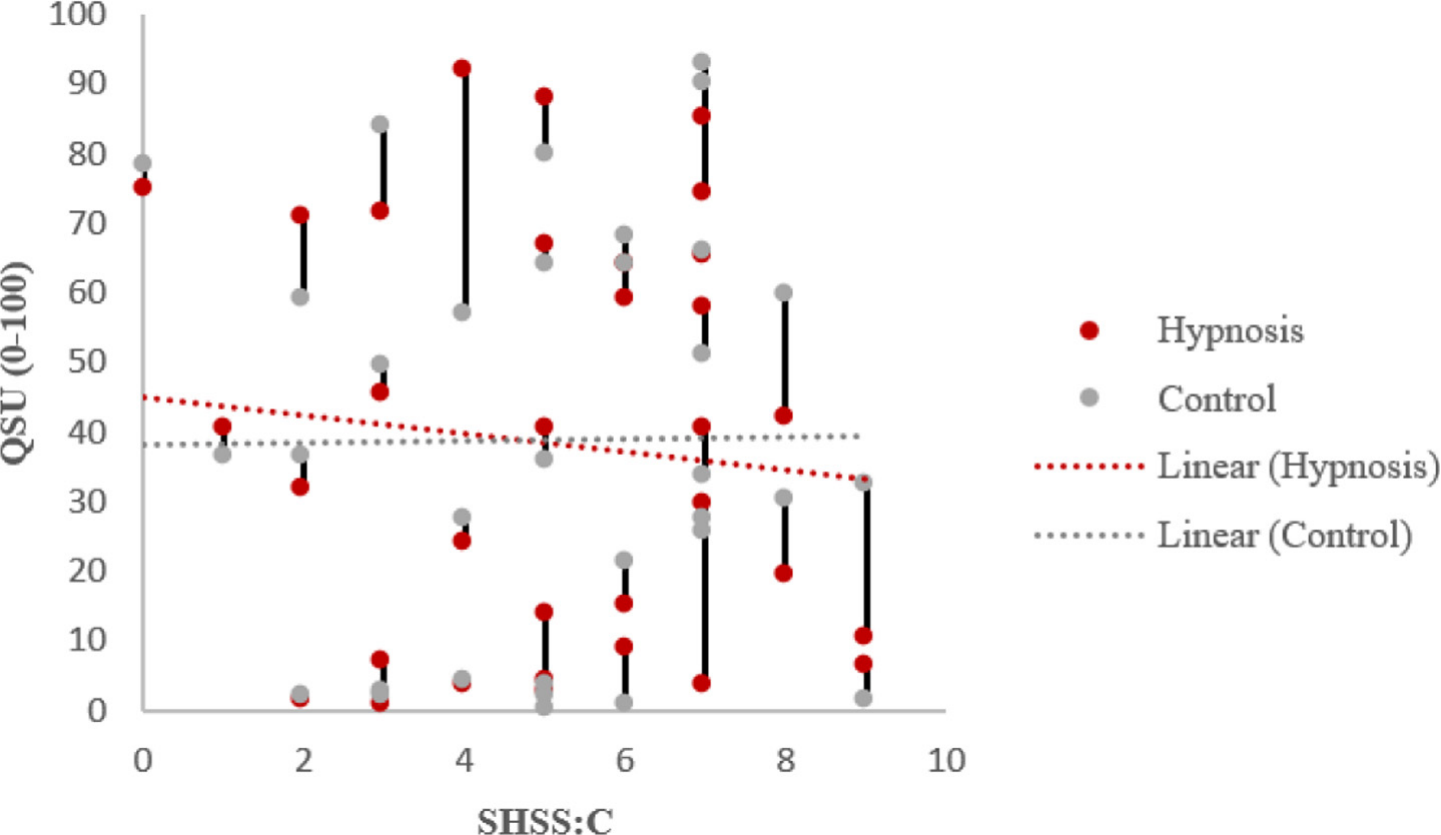
Craving as a function of hypnotic susceptibility (vertical lines connect data from same subject).

Table 2 reveals that the Susceptibility × Suggestion interaction was not significant for the classic Stroop effect or the smoking Stroop effect. Contrary to hypothesis, there was no evidence that participants with higher SHSS:C scores exhibited a greater decrease in the classic Stroop effect or smoking Stroop effect during hypnotic suggestion (vs. control) than participants with lower SHSS:C scores (Table 2). Table 2 also reveals that the Susceptibility × Suggestion interaction was significant for QSU ratings, suggesting that participants with higher SHSS:C scores exhibited a greater decrease in craving ratings during hypnotic suggestion (vs. control) than participants with lower SHSS:C scores (Fig. 2). Hypnotic suggestion significantly reduced QSU ratings in participants with SHSS:C scores ≥ 7. There was no evidence for an effect of Suggestion in participants with low SHSS:C scores (≤2), *F*(1, 3) = 1.23, *p* = 0.35 (not shown in Table 2).

**Table 2:** Results of GLMs.

			**Condition**				**Susceptibility**				**Susceptibility × Condition**			
**DV ↓**	Model	*n*	*df*	*F*	*p*	*ES*	*df*	*F*	*p*	*ES*	*df*	*F*	*p*	*ES*
Classic Stroop	1	33	1, 30	0.01	0.94	0.000	1, 30	1.42	0.24	0.045	1, 30	0.01	0.91	0.000
	2	4	1, 2	0.55	0.53	0.217	n/a	n/a	n/a	n/a	n/a	n/a	n/a	n/a
	3	11	1, 9	0.73	0.73	0.075	n/a	n/a	n/a	n/a	n/a	n/a	n/a	n/a
Smoking Stroop	1	32	1, 29	0.26	0.62	0.009	1, 29	0.03	0.87	0.001	1, 29	0.02	0.89	0.001
	2	4	1, 2	6.27	0.13	0.758	n/a	n/a	n/a	n/a	n/a	n/a	n/a	n/a
	3	11	1, 9	0.35	0.57	0.037	n/a	n/a	n/a	n/a	n/a	n/a	n/a	n/a
QSU	1	33	1, 30	0.14	0.71	0.004	1, 30	0.026	0.873	0.001	1, 30	4.24	0.048	0.124
	2	4	1, 2	11.12	0.08	0.848	n/a	n/a	n/a	n/a	n/a	n/a	n/a	n/a
	3	11	1, 9	12.41	0.007	0.580	n/a	n/a	n/a	n/a	n/a	n/a	n/a	n/a

Table Note: *n* = number of subjects; *F* = F value from GLM; Order is included as a between-subject factor; ES = Effect Size (Partial Eta Squared). Order is included as categorical variable in models (effects of Order not shown); Model = 1 is the full GLM with all subjects; the Susceptibility × Condition interaction is the main result of interest; Model = 2 is a GLM testing the effect of Suggestion on study outcomes for the 4 subjects with SHSS:C scores ≥ 8; Model = 3 is a GLM testing the effect of Suggestion on study outcomes for the 11 subjects with SHSS:C scores ≥ 7.

## 4 Discussion

The main results of this study were as follows. First, contrary to our hypotheses, there was no evidence that hypnotic susceptibility moderated the effect of Suggestion on the classic Stroop or smoking Stroop effect. Second, in individuals with high levels of hypnotic susceptibility, there was no evidence that the classic Stroop effect or the smoking Stroop effects were reduced by hypnotic suggestion. Third, susceptibility significantly moderated the effect of hypnotic suggestion on craving, such that hypnotic suggestion reduced craving in individuals with high levels of hypnotic susceptibility.

For the classic Stroop effect, the lack of effect observed in the current study as opposed to the previous studies could be due to a number of reasons. First, the current study used a lower threshold for high hypnotic susceptibility: it used “≥8” and “≥7” as cut-offs, whereas most studies used “≥10” or “≥11”. Second, whereas other studies generally used student volunteers, the current study recruited an older, predominantly African American sample. Contemporary theories of hypnosis state that expectations of hypnosis’ efficacy predict the degree of response (Kirsch & Lynn, 1999). In this study, subjects may have expected hypnosis to have an impact on craving due to its well-known use as a method to quit smoking, but participants may not have thought that hypnosis would influence performance on a computer task such as the classic and smoking Stroop. Participants in previous studies may have held expectations that hypnosis could change cognition due to higher levels of education.

Last, it is possible that in the hypnotic suggestion condition participants may have experienced reduced frontal lobe function so that top-down control would be compromised, preventing the reduction of Stroop interference (see Parris, 2017). Further reseach can experimentally manipulate procedural features of the hypnotic suggestion to examine the effect of these methodological factors on study outcomes. For example, in Raz et al. (2002) participants were dehypnotized between administration of the suggestion and completion of the Stroop tasks (email communication, 09/09/19), which may account for differences in outcomes on Stroop measures with those documented in the current paper. Regardless, in future studies it is crucial that the procedures used are comprehensively reported to permit researchers to examine the influence of these methodological variables.

The classic Stroop task was approximately 6 min in duration (on average). It is possible that practice on the classic Stroop task (which was always completed before the smoking Stroop task) served to reduce the smoking Stroop effect in both conditions. The participants could have been “over-practiced” in performing Stroop tasks which distorted data in the smoking Stroop. For example, participants may have adopted strategies to try to minimize the distracting effect of the incongruent words, such as trying to focus on individual letters. In any case, the absence of a robust smoking Stroop effect in the control condition may have made it more difficult to reduce the smoking Stroop effect during the hypnotic suggestion condition.

The most interesting finding of the study was that susceptibility moderated the effect of hypnotic suggestion on craving ratings, such that individuals with SHSS:C scores ≥ 7 reported lower craving in the hypnotic suggestion condition than in the control condition. Therefore, an assessment of hypnotic susceptibility can help identify smokers whose craving can be reduced, at least acutely, by hypnotic suggestion. Given that hypnotic suggestion had no significant effect on the smoking Stroop (or classic Stroop) effect, the effect of hypnotic suggestion on craving may be independent of any effect of hypnotic suggestion on cognitive processes. That said, it is possible that hypnotic suggestion does influence cognitive processes that underlie craving, but the assessments used in the current study were insufficiently sensitive to detect an effect. Also, note that no suggestions were given for decreasing craving, only suggestions for changing performance during the Stroop tasks. The hypnotic induction and/or suggestions used could have reduced craving through other mechanisms, such as reducing stress. That is, the hypnotic induction and deepening of the SHSS:C (which includes suggestions for deep relaxation and hypnotic sleep), may have reduced stress and subsequent decreased cravings. This could not be tested due to non-inclusion of a measure of stress.

Participants reported higher levels of side effects in the hypnotic suggestion condition than the control condition (Section S.5, Supplementary Materials). However, these side effects are unlikely to mediate the effect of Suggestion on craving because there was no interaction between Susceptibility and Suggestion for side effects.

The strengths of the study included the following. This study examined the effect of hypnotic suggestion on an addiction Stroop task and on craving (several previous studies focused solely on the effect of hypnotic suggestion on performance on the classic Stroop task). Second, a community sample was recruited, permitting an assessment of the generalizability of findings that have been observed in university settings.

The most important limitation of the current study was the relatively small sample size. That said, if the data reported by Raz et al. (2002) reflect the true effect size in the population the study had power = 0.98 to detect the pertinent interaction (see Supplementary Materials Section S.2). Note, however, that there is evidence that effect sizes reported in the literature may be over-estimates of the “true” effect sizes (Open Science Collaboration, 2015). A second limitation is that a smaller proportion of participants (12.12%) than expected (20.00%) scored in the highly susceptible range. This limitation seems particularly relevant to analysis of the smoking Stroop data, as the data appeared to indicate that the effect of hypnotic induction tended in right direction (though, of course, statistical significance was not obtained). Other, limitations included the following. As noted earlier, the order of completion of the classic and smoking Stroop tasks was not counterbalanced. There was variability in recency of smoking as assessed by carbon monoxide levels at the beginning of the study session which could have affected cognition and craving. Unfortunately, recency of smoking prior to the session was not assessed using self-report, and nicotine withdrawal symptoms were also not assessed. In addition, degree of sleepiness or level of caffeine intake could have influenced cognition and susceptibility to hypnosis, and data on these and other “state” variables were not collected. Finally, the within-subject design permits the possibility of carry-over effects, which can complicate interpretation of data.

Future research should examine the effect of hypnotic suggestion on cognition and craving in larger samples of highly susceptible smokers to obtain a more precise estimate of the effect of hypnotic suggestion on cognition and craving in smokers. Future research could also examine if hypnotic suggestion can exert durable changes in craving and test different “types” of hypnotic suggestion to determine the suggestion types that are most effective in reducing craving. As stated above, the suggestions used in this study targeted cognitive (semantic) processing, whereas Li et al. (2017) used an aversive hypnotic suggestion procedure. The effect of other types of hypnotic suggestions on craving needs further exploration. A future study could examine if craving differs after: suggestions targeting relaxation only; aversive suggestions (similar to Li et al., 2017); suggestions targeting cognition; and suggestions directly targeting specific aspects of craving. One could also test if formal hypnotic inductions are needed. Finally, if craving is malleable in a subset of smokers, hypnotic suggestion-based treatments may be developed for high susceptible individuals.

## Declaration of Competing Interest

All authors declare no conflicts of interest.
