# Supplementary material for: Effect of hypnotic suggestion on cognition and craving in smokers
Source: Addict Behav Rep. 2019 Nov 26;11:100220. doi: 10.1016/j.abrep.2019.100220 (PMC6926110; doi:10.1016/j.abrep.2019.100220)
Supplement: Supplementary data 1 [file mmc1.docx]

**Supplementary Materials**

**S.1 Stroop Tasks**

**Data Processing**

On the Stroop tasks, reaction times (RTs) from all incorrect responses were discarded, as were RTs less than 100 milliseconds. The classic Stroop has three scores derived from average RTs for each trial type: incongruent minus congruent (classic Stroop effect), incongruent minus neutral, and congruent minus neutral. The classic Stroop effect was the primary dependent variable in this study. The smoking Stroop effect was computed as smoking minus neutral.

**Reliability**

For the control condition, the estimated internal reliabilities (using the Spearman-Brown formula for odd vs even trials) of mean RT for the incongruent and congruent trials in the classic Stroop were excellent (*r* = .96 & .98, respectively). The estimated internal reliability for the classic Stroop effect (the difference score) was *r* = .54. For the hypnotic suggestion condition, the estimated internal reliabilities of mean RT for the incongruent and congruent trials in the classic Stroop were excellent (*r* = .94 & .97, respectively). The estimated internal reliability for the classic Stroop effect was *r* = .64.

For the smoking Stroop task, for the control condition the estimated internal reliabilities (using the Spearman-Brown formula for odd vs even trials) of mean RT for the smoking and neutral trials in the smoking Stroop were excellent (*r* = .97 & .97, respectively). The estimated internal reliability for the smoking Stroop effect (the difference score) was *r* = .80. For the hypnotic suggestion condition, the estimated internal reliability of mean RT for the smoking and neutral trials in the smoking Stroop were excellent (*r* = .95 & .92, respectively). The estimated internal reliability for the smoking Stroop effect was *r* = .44.

**Summary Statistics**

On the Stroop tasks, for the control condition the mean reaction times for each condition were: incongruent trials = 889.12 ms (SD = 168.03), congruent trials = 776.06 ms (SD = 138.26), smoking trials = 806.63 ms (SD = 154.58), and neutral trials (smoking Stroop) = 801.12 ms (SD = 135.36). For the hypnotic suggestion condition, the mean reaction times were: incongruent trials = 899.87 ms (SD = 152.49 ms), congruent trials = 786.79 ms (SD = 134.64), smoking trials = 831.91 ms (SD =151.50), and neutral trials (smoking Stroop) = 833.57 ms (SD = 123.69).

Over all participants, the mean classic Stroop effect (*N*=33) was 113.05 ms (*SD* = 65.47) and 113.08 ms (*SD* = 71.09) on the control and hypnotic suggestion conditions respectively. The mean smoking Stroop effect (*n*=32) was 5.51 ms (*SD* = 59.08) and -1.66 ms (*SD* = 71.47) on the control and hypnotic suggestion conditions respectively. The mean QSU ratings (*N*=33) were 38.99 (*SD* = 29.66) and 38.22 (*SD* = 29.89) on the control and hypnotic suggestion conditions respectively.

Following Raz et al. (2002), we also recomputed all analyses when also excluding RTs that were 3 *SD*s either above or below the mean on each task at each administration (first and second). In total, 4.7% and 3.8% of data were excluded from the classic Stroop and smoking Stroop tasks respectively (including exclusions for incorrect responses). Results from these analyses are presented in Table S1, and they yield the same conclusions as results from analyses presented in the paper.

**S.2 Power Analysis**

All tests used were 2-tailed, with alpha value set to .05. Power analyses were performed using G Power Version 3 (Faul, Erdfelder, Lang, & Buchner, 2007), and were based on the effect size for the Susceptibility x Suggestion interaction presented in Raz et al. (2002). The *F* value reported in Raz et al. for the pertinent Group x Suggestion interaction term for the incongruent-congruent trials, *F*(1,28)=17.13 (corresponding to a *t* value of 4.14) was converted to an *r* value (*r* = .62, i.e., a large effect sizes). With *N* = 33 and *r* = .62, the study had estimated power = .98 to detect an interaction between Susceptibility (entered as a continuous variable) and Suggestion (a within-subjects variable with 2 levels: hypnotic suggestion vs control). For testing the effect of Suggestion on study outcomes, assuming 10 highly susceptible individuals, the study had power = .80 to detect a difference (between Suggestion conditions) in the Stroop effect of *d* = 1.00. Based on the *F* value presented in Raz et al., *F* (1, 30) = 29.25, the estimated effect size for this within-subject comparison is *d* = 1.96, and therefore larger than *d* = 1.00.

**S.3 Analytic Plan**

**Covariates**

Gender (coded as 1=male, 2=female) was not significantly associated with SHSS:C (*r* = -.12, *p* = .49). Additionally, there was no evidence that age *(r* = -.27, *p* = .12), race (coded as 0=Other, 1=AA) (*r* = -.27, *p* = .12), years of education (*r* = .02, *p* = .89), or income (*r* = .25, *p* = .16) were associated with SHSS:C scores. Therefore, gender, age, race, years of education, and income were not included as covariates. Finally, as noted, subjects attended the lab session at different times of day. There was no evidence that time of day was associated with Susceptibility (*r* = -0.06, *p* = .76).

Additionally, the association between gender, age, race, years of education, and income and study dependent variables was examined. Gender was not significantly associated with the classic Stroop effect in the hypnotic suggestion (*r* = .10, *p* = .57) or control condition (*r* = .08, *p* = .65), or with the smoking Stroop effect in the hypnotic suggestion (*r* = -.07, *p* = .70) or control condition (*r* = -.11, *p* = .54), or with QSU ratings in the hypnotic suggestion (*r* = -.08, *p* = .65) or control condition (*r* = -.20, *p* = .27). Age, race (binary), and income were also not significantly associated with the classic Stroop effect in the hypnotic suggestion or control condition, or with the smoking Stroop effect in the hypnotic suggestion or control condition, or with QSU ratings in the hypnotic suggestion or control condition (all *p*s > .13, .21, and .32 for age, race, and income respectively). Years of education was correlated with the smoking Stroop effect in the control condition (*p* = .01), but not the other variables (all *p*s > .14). Inclusion of years of education (centered) as a covariate in analysis of the smoking Stroop effect did not change any of the findings.

Regarding smoking variables, there was no evidence that FTND scores (*r* = 0.02, *p* = .91) or expired breath CO level (*r* = 0.09, *p* = .63) were associated with Susceptibility. There was also no evidence that FTND scores were associated with the classic Stroop, smoking Stroop, or QSU ratings in the hypnotic suggestion condition (all *p*s > .18) or the Control condition (all *p*s > .16). Likewise, there was also no evidence that expired breath CO levels were associated with the classic Stroop, smoking Stroop, or QSU ratings in the hypnotic suggestion condition (all *p*s > .63) or the Control condition (all ps > .17).

**S.4 Effects of Order**

Seventeen subjects completed the Control-Hypnosis condition, and 16 completed the Hypnosis-Control condition. Order of completion (0=Control-Hypnosis, 1=Hypnosis-Control) was not correlated with SHSS:C, *r*(31) = .10, *p* = .60. As noted earlier, Order was included as a variable in all GLMs.

Participants in the Control-Hypnosis condition had a classic Stroop effect of 127.50 ms (*SD* = 75.03) in the control condition (the first condition they completed), and a classic Stroop effect of 99.49 ms (*SD* = 62.52) in the hypnotic suggestion condition (the second condition they completed). Participants in the Hypnosis-Control condition had a classic Stroop effect of 127.52 ms (*SD* = 78.62) in the hypnotic suggestion condition (the first condition they completed), and a classic Stroop effect of 97.71 ms (*SD* = 51.50) in the control condition (the second condition they completed). Therefore, participants tended to have a higher classic Stroop effect in the condition they performed first, consistent with the idea that subjects’ performance improves with practice (i.e., subjects exhibit smaller classic Stroop effects over repeated assessments). This is reflected in the presence of a significant Suggestion x Order interaction, *F*(1, 30) = 5.06, *p* = .03.

In contrast with the results for the classic Stroop effect, there was no evidence for a Suggestion x Order interaction for analysis of the smoking Stroop effect, *F*(1, 29) = 0.00, *p* = .97.

Participants in the Control-Hypnosis condition had a mean QSU rating of 45.88 (*SD* = 31.00) in the control condition (the first condition they completed), and a mean QSU rating of 41.59 (*SD* = 33.15) in the hypnotic suggestion condition (the second condition they completed). Participants in the Hypnosis-Control condition had a mean QSU rating of 34.63 (*SD* = 26.59) in the hypnotic suggestion condition (the first condition they completed), and a mean QSU rating of 31.66 (*SD* = 27.21) in the control condition (the second condition they completed). Therefore, participants tended to report more craving in the condition they performed first, consistent with the idea that craving declines during the course of the study. This is reflected in the presence of a significant Suggestion x Order interaction, *F*(1, 30) = 4.24, *p* = .04, in the analysis of craving data.

**S.5 Side Effects**

The presence of a side effect was defined by ≥1 point increase in the side effect when compared to the most proximal assessment before hypnosis was administered. Using this definition, side effects due to hypnosis were fairly common (54.45% of all subjects reported at least 1 side effect). The most common side effect (as defined by the change score above) was “drowsiness” (48.48%). The next most common side effect (as defined by the change score) was “confusion” (18.18% of participants). “Headache” was reported by one subject (3.03% of the sample). Side effects were also mild. In the hypnosis condition, no side effect item was rated with an (absolute) value of 3 [“a lot”] or 4 [“very much”] (on a 0-4 scale); all side effect items were either rated as a 1 [“very little”] or 2 [“somewhat”].

A GLM examined the effect of Susceptibility and Suggestion on side effects (mean of 3 items, which all used a 0-4 scale). The main effect of Suggestion was significant, *F*(1, 30) = 14.64, *p* < .001, meaning that there was evidence that side effects were higher in the hypnotic suggestion condition (*M* = 0.41, *SD* = 0.39) than the control condition (*M* = 0.18, *SD* = 0.24). There was no evidence that the effect of Suggestion was moderated by Susceptibility, *F*(1, 30) = 0.57, *p* = .46.

*Table S1*. Results of GLMs (Exclusions of Reaction Time Outliers)

|  |  |  |  | **Condition** | | | |  | **Susceptibility** | | | |  | **Susceptibility x Condition** | | | |
| --- | --- | --- | --- | --- | --- | --- | --- | --- | --- | --- | --- | --- | --- | --- | --- | --- | --- |
| **DV ↓** | Model | *n* |  | *df* | *F* | *p* | *ES* |  | *df* | *F* | *p* | *ES* |  | *df* | *F* | *p* | *ES* |
|  |  |  |  |  |  |  |  |  |  |  |  |  |  |  |  |  |  |
| Classic Stroop | 1 | 33 |  | 1, 30 | 0.44 | .51 | .015 |  | 1, 30 | 0.22 | .64 | 0.007 |  | 1, 30 | 0.11 | .74 | .004 |
|  | 2 | 4 |  | 1, 2 | 3.76 | .19 | .653 |  | n/a | n/a | n/a | n/a |  | n/a | n/a | n/a | n/a |
|  | 3 | 11 |  | 1, 9 | 0.01 | .92 | .001 |  | n/a | n/a | n/a | n/a |  | n/a | n/a | n/a | n/a |
| Smoking Stroop | 1 | 32 |  | 1, 29 | 1.39 | .25 | .046 |  | 1, 29 | 0.21 | .65 | .007 |  | 1, 29 | 0.10 | .75 | .003 |
|  | 2 | 4 |  | 1, 2 | 3.42 | .21 | .631 |  | n/a | n/a | n/a | n/a |  | n/a | n/a | n/a | n/a |
|  | 3 | 11 |  | 1, 9 | 0.15 | .70 | ,016 |  | n/a | n/a | n/a | n/a |  | n/a | n/a | n/a | n/a |

Table S1 Note: Results from GLMs after exclusion of RTs 3 *SD*s either above or below the mean on each task at each administration. *n* = number of subjects; *F* = F value from GLM; *ES* = Effect Size (Partial Eta Squared). Order is included as a between-subject factor. Order is included as categorical variable in models (effects of Order not shown); Model = 1 is the full GLM with all subjects; the Susceptibility x Condition interaction is the main result of interest; Model = 2 is a GLM testing the effect of Suggestion on study outcomes for the 4 subjects with SHSS:C scores ≥ 8; Model = 3 is a GLM testing the effect of Suggestion on study outcomes for the 11 subjects with SHSS:C scores ≥ 7.


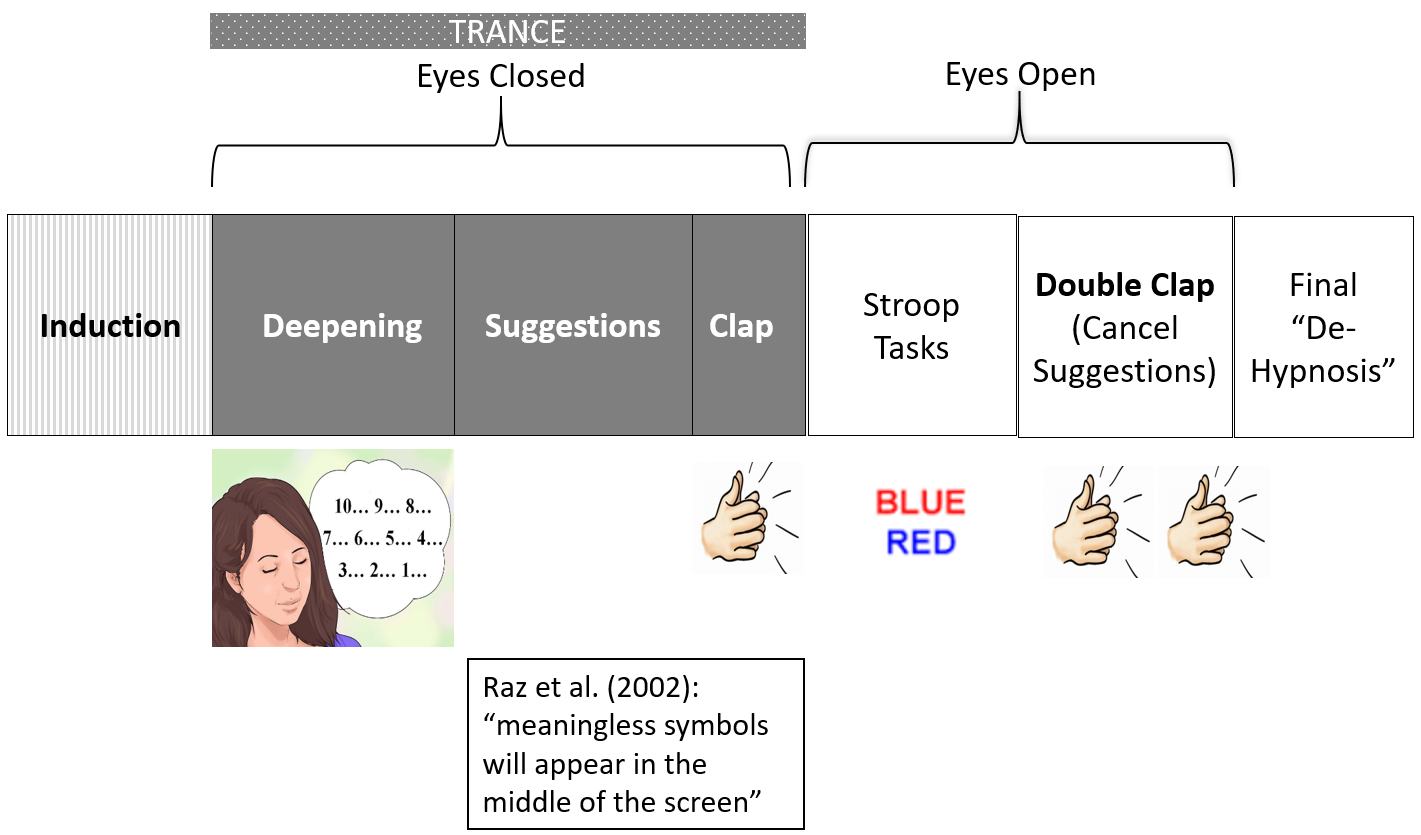


Figure S1
